# Electrocatalytic oxygen reduction activity of AgCoCu oxides on reduced graphene oxide in alkaline media

**DOI:** 10.3762/bjnano.13.89

**Published:** 2022-09-26

**Authors:** Iyyappan Madakannu, Indrajit Patil, Bhalchandra Kakade, Kasibhatta Kumara Ramanatha Datta

**Affiliations:** 1 Functional Nanomaterials Laboratory, Department of Chemistry, Faculty of Engineering and Technology, SRM Institute of Science and Technology, Kattankulathur – 603203, Tamil Nadu, India; 2 Department of Chemistry, Faculty of Engineering and Technology, SRM Institute of Science and Technology, Kattankulathur – 603203, Tamil Nadu, India

**Keywords:** copper cobalt oxide NPs, microwave synthesis, oxygen electroreduction, reduced graphene oxide, silver NPs

## Abstract

Silver-based electrocatalysts as promising substitutes for platinum materials for cathodic oxygen electroreduction have been extensively researched. Electrocatalytic enhancement of the Ag nanoarchitectonics can be obtained via support structures and amalgamating Ag with one or two additional metals. The work presented here deals with a facile microwave-assisted synthesis to produce bimetallic Ag-Cu and Ag-Co (1:1) oxide nanoparticles (NPs) and trimetallic AgCuCo (0.6:1.5:1.5, 2:1:1, and 6:1:1) oxide NPs supported on a reduced graphene oxide (rGO) matrix. Morphology, composition, and functional groups were methodically analysed using various microscopic and spectroscopic techniques. The as-prepared electrocatalysts were employed as cathode substrates for the oxygen reduction reaction (ORR) in alkaline medium. Varying the Ag fraction in copper cobalt oxide has a significant influence on the ORR activity. At a ratio of 2:1:1, AgCuCo oxide NPs on rGO displayed the best values for onset potential, half-wave potential, and limiting current density (*J*_k_) of 0.94 V vs RHE, 0.78 V, and 3.6 mA·cm^−2^, respectively, with an electrochemical active surface area of 66.92 m^2^·g^−1^ and a mass activity of 40.55 mA·mg^−1^. The optimum electrocatalyst shows considerable electrochemical stability over 10,000 cycles in 0.1 M KOH solution.

## Introduction

Fuel cells and rechargeable metal–air batteries have become an integral part of the renewable energy system because of their superior efficiency, high power density, and reliability. Also, they are environmentally friendly with zero emissions at the time of use. These systems have the ability to convert chemical energy into electric energy with the highest conversion possible [1–2]. The active electrode reactions include the hydrogen oxidation reaction (HOR) and the oxygen reduction reaction (ORR). The slow reaction rates of the electrode processes impede the efficiency and, thus, require innovative catalyst designs. The ORR is an irreversible, complex (involving multiple steps and intermediates O, OH^−^, O^2−^, HO^2−^ and H_2_O_2_) and kinetically slow process (via two- or four-electron transfer) dominating the overall performance of the reaction [3–4]. There is an increasing use of platinum catalysts with diverse morphologies and the combination with noble and non-noble metal-based alloy/multimetallic nanoparticles (NPs) as potential electrocatalysts under extreme pH values [5–14]. In particular, the ORR in alkaline environments with faster kinetics and lower over potential requires stable transition metal-derived electrocatalysts [15]. The major hurdles for Pt-based ORR electrode catalysts in alkaline media include high cost, low operational stability, fuel crossover effects, and carbon monoxide poisoning [16–17].

The electrocatalytic reactivity (mechanism and kinetics) of silver has similarities to that of Pt regarding the ORR performance, with considerably high onset potential, half-wave potential, current density, and number of transferred electrons. The important attributes include high electrical conductivity, cost-effectiveness (50 times lower than Pt), and the ability to execute the ORR via a single step (four-electron transfer). Thus, Ag and its bi- and trimetallic alloys, with and without supporting matrices, have been extensively researched as potential ORR electrocatalysts [14,18–19]. Oxophilicity, agglomeration, and poor chemical stability of Ag require the amalgamation of Ag with other metals for a better optical and catalytic activity [20]. Chen et al. synthesised Ag nanoscale alloys containing metals such as copper, cobalt, iron, and indium via pulse film electrodeposition. Among the combinations, the Ag–Cu (3:1) alloy showed the better electrode catalytic activity and the highest onset (0.85 V vs RHE) and half-wave potential (0.76 V vs RHE) with a limiting current density of 4.19 mA·cm^−2^, along with an electron transfer value of 3.86 in 0.1 M KOH [21]. Linic and co-workers reported the ORR activity of Ag–Co NPs dispersed on Vulcan XC72 carbon by incipient-wetness impregnation [22]. In general, the addition of a third metal to a bimetallic composition is considered to be an effective method to augment the absorption energy and improve the kinetics of the ORR [23]. Gu et al. prepared supportless Ag nanowires and 1D mesoporous hollow AgPdPt nanotubes by micelle-assisted galvanic replacement followed by acid etching. They found that hollow AgPdPt structures exhibited a better ORR activity with onset and half-wave potential of 0.99 V and 0.90 V vs RHE, respectively, and a limiting current density of 5.3 mA·cm^−2^ in 0.1 M KOH with an electron transfer value of 3.97 [24]. The strong interactions among the unsupported trimetallic nanoparticles leads to aggregation resulting in reduced activity and stability. In this regard, structural regulation can be obtained via atomic-level manipulation using established materials chemistry concepts towards the assembly of functional nanoarchitectonics [25–27]. The assembly of nanoscale objects through combination and in situ growth routes, leading to high-performance nanoarchitectonics, is an interesting strategy. An interesting choice could be the support/assembly of trimetallic AgCuCo particles via in situ formation integrated with conductive (graphitic) supports to further enhance the electrocatalytic properties regarding the ORR in alkaline medium.

We designed trimetallic AgCuCo oxide NPs supported on rGO using a microwave-assisted approach with different fractions of Ag, Cu, and Co, that is, Ag_0.6_Co_1.5_Cu_1.5_ (ACC-1), Ag_2.0_Co_1.0_Cu_1.0_ (ACC-2), and Ag_6.0_Co_1.0_Cu_1.0_ (ACC-3). Our method is convenient and efficient for designing a sustainable electrode material for the ORR in alkaline media. XRD, FTIR, and SEM were used to analyse formation and morphology of all synthesized electrocatalysts. Also, the ORR activity in 0.1 M KOH was investigated. Among all samples, ACC-2 exhibited the best onset potential, half-wave potential, and limiting current density (*J*_k_) of 0.94 and 0.78 V vs RHE and 3.6 mA·cm^−2^, respectively, with an electrochemical active surface area (ECSA) of 66.92 m^2^·g^−1^ and a mass activity of 40.55 mA·mg^−1^. We investigated bonding nature, composition, and morphology of the ACC-2 sample using XPS and TEM. The material is electrochemically stable up to 10,000 potential cycles, highlighting the durability of the electrocatalyst.

## Results and Discussion

**Figure 1:**
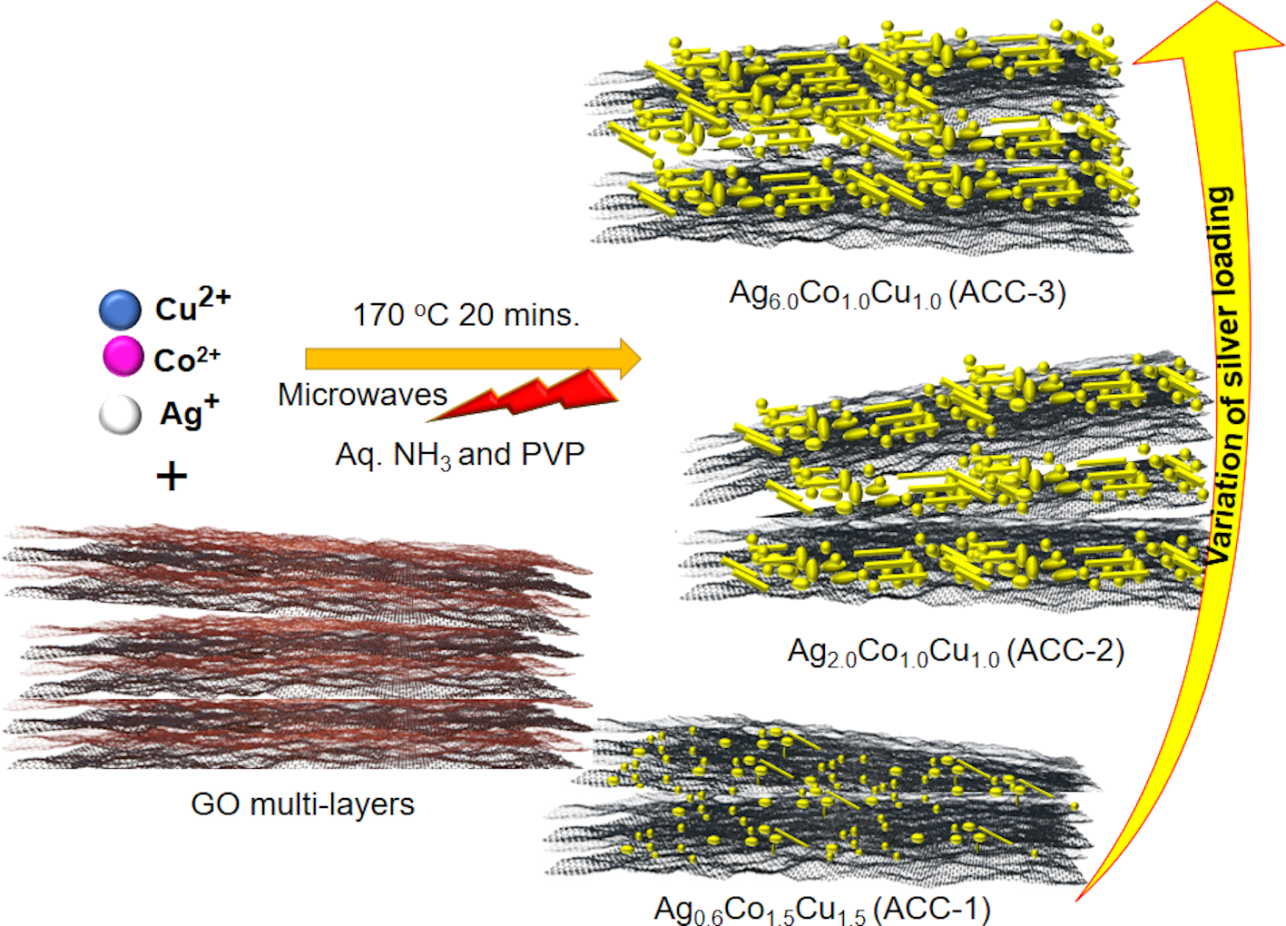
Illustration of the formation of AgCoCu oxide NPs over rGO with tuneable Ag fractions.

The microwave-assisted one-pot synthesis connecting graphene oxide (GO) nanosheets with bi- and trimetallic precursors under alkaline conditions is presented in Figure 1. GO serves as functional network and conducting matrix, while PVP acts as structure-directing agent, leading to the formation of the bimetallic (Ag-CuO and Ag-Co_3_O_4_) and trimetallic (AgCuCo oxide, i.e., ACC-1, ACC-2, and ACC-3) assemblies on rGO. The self-assembled nanoarchitectonics were henceforth utilized as electrode materials for the ORR reaction in alkaline medium. The rationale behind the selection of Cu and Co is their similar crystal structure to that of silver, besides their catalytic oxidizing ability. Moreover, to date, there are no reports on the evaluation of the electrochemical ORR activity in alkaline media employing AgCuCo oxides supported on rGO.

**Figure 2:**
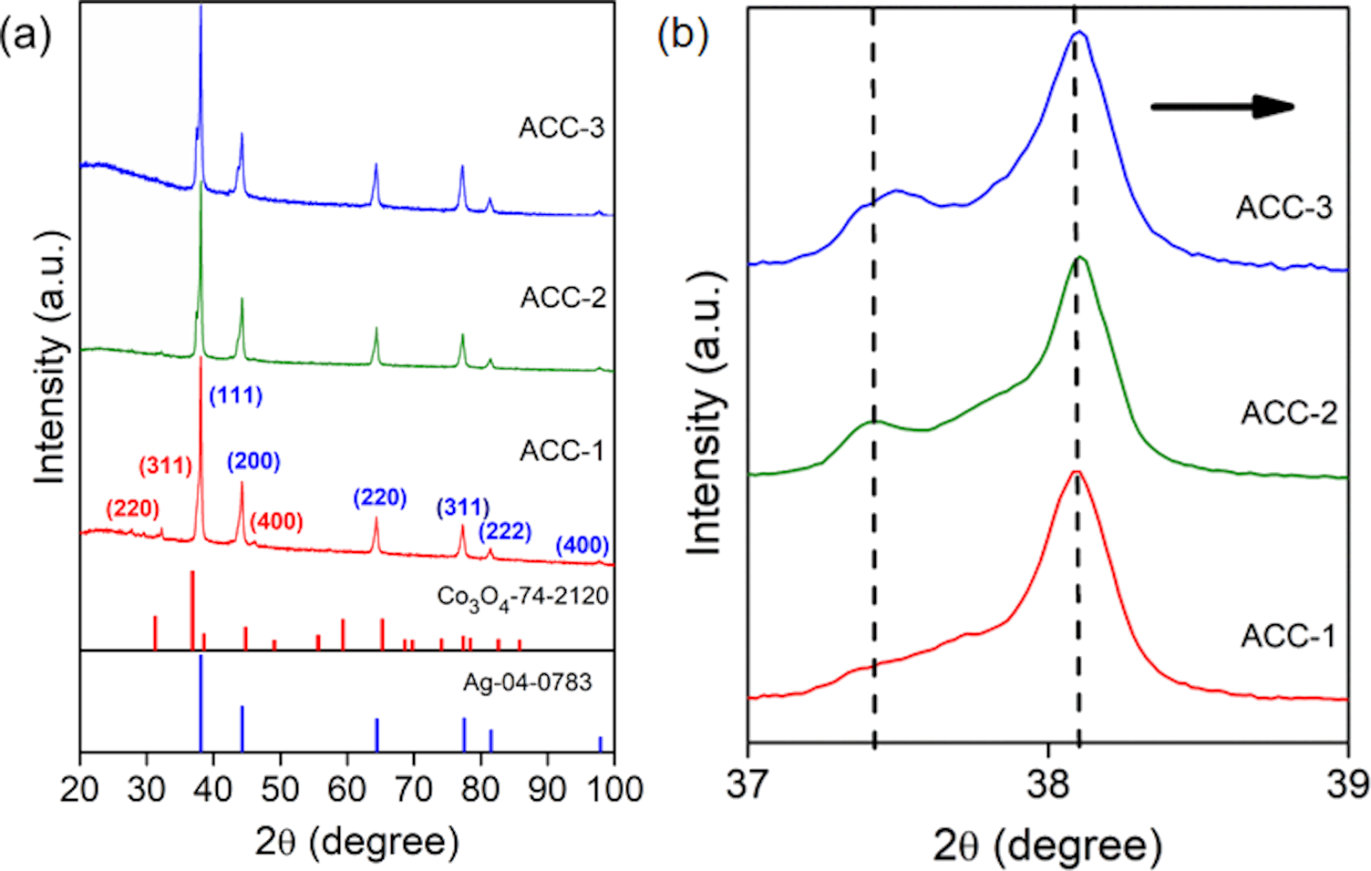
(a) PXRD patterns of ACC-1, ACC-2, and ACC-3. (b) Magnified area displaying angle shifts in the range of 2θ = 37°–39°.

To identify the phase formation and crystallinity of the assembled binary and ternary metallic oxide NPs, powder X-ray diffraction (PXRD) measurements were carried out. The PXRD patterns of the binary and ternary NPs on rGO showed peaks located at 2θ = 38.1°, 44.2°, 64.3°, and 77.1°, which could be indexed to the (111), (200), (220), and (311) planes, respectively, of fcc Ag (JCPDS #04-0783) (Figure 2a and Figure S1, Supporting Information File 1). Upon closer examination of the trimetallic AgCoCu system, we observe that the (111) reflection is slightly shifted towards higher 2θ angles with increasing Ag content compared to the bimetallic counterpart, indicating the incorporation of Cu and Co atoms in the Ag lattice (Figure 2b). Additionally, we observe minor peaks related to Co oxide (2θ = 32.3°, 37.6°, and 45.8° attributed to the (220), (311), and (400) planes, respectively, of Co_3_O_4_ [JCPDS #74-2120]), underlining the integration of Co atoms in the Ag lattice [28–29], while reflections of Cu mask the Ag reflections. We see a systematic variation of the particle sizes for the bi- and trimetallic NPs (Table S1, Supporting Information File 1), which could be connected to the intermixing and phase segregation of mainly Co_3_O_4_ in the trimetallic oxide NPs.

The graphene oxide support provides the necessary functional groups for the cohesion of binary and ternary metallic oxide NPs (Figure S2a, Supporting Information File 1). The functional groups and the metal oxygen bonds were determined by FTIR spectroscopy, besides the slight changes of the peak positions by varying the silver fraction in the trimetallic catalysts (Figure S2a,b, Supporting Information File 1). The surface morphology of rGO-supported bimetallic (Ag-CuO and Ag-Co_3_O_4_) and trimetallic (ACC-1, ACC-2 and ACC-3) NPs, as well as the supportless ACC-2*, were analysed through scanning electron microscope (SEM). For the bimetallic NPs supported on rGO, we observe spherical and triangular particles distributed over few-layered rGO nanosheets. The uniform distribution of the bimetallic NPs over the edges and the surface of the rGO highlights the importance of the functional layered scaffold anchoring metal NPs (Figure S3a,b, Supporting Information File 1). Likewise, for the trimetallic NPs anchored on the rGO support, we observe various sizes and morphologies on multilayered rGO nanosheets. The variation in the morphology stems from the fusion of Cu and Co into Ag nanostructures. Also, upon increasing the Ag fraction in the sample, the dispersion of the trimetallic assembly considerably increases, which is clearly reflected in Figure S3c–e, Supporting Information File 1. The importance of the rGO support is further evident from the imaging of supportless the ACC-2* sample, wherein we notice irregular morphologies besides agglomeration of the particles (Figure S3f, Supporting Information File 1). The increasing silver fraction in the trimetallic assemblies was observed from EDX analysis as shown in Figure S4, Supporting Information File 1. After examining the composition of bi- and trimetallic assemblies on rGO, the electrochemical ORR performance parameters of these materials in alkaline medium were investigated.

**Figure 3:**
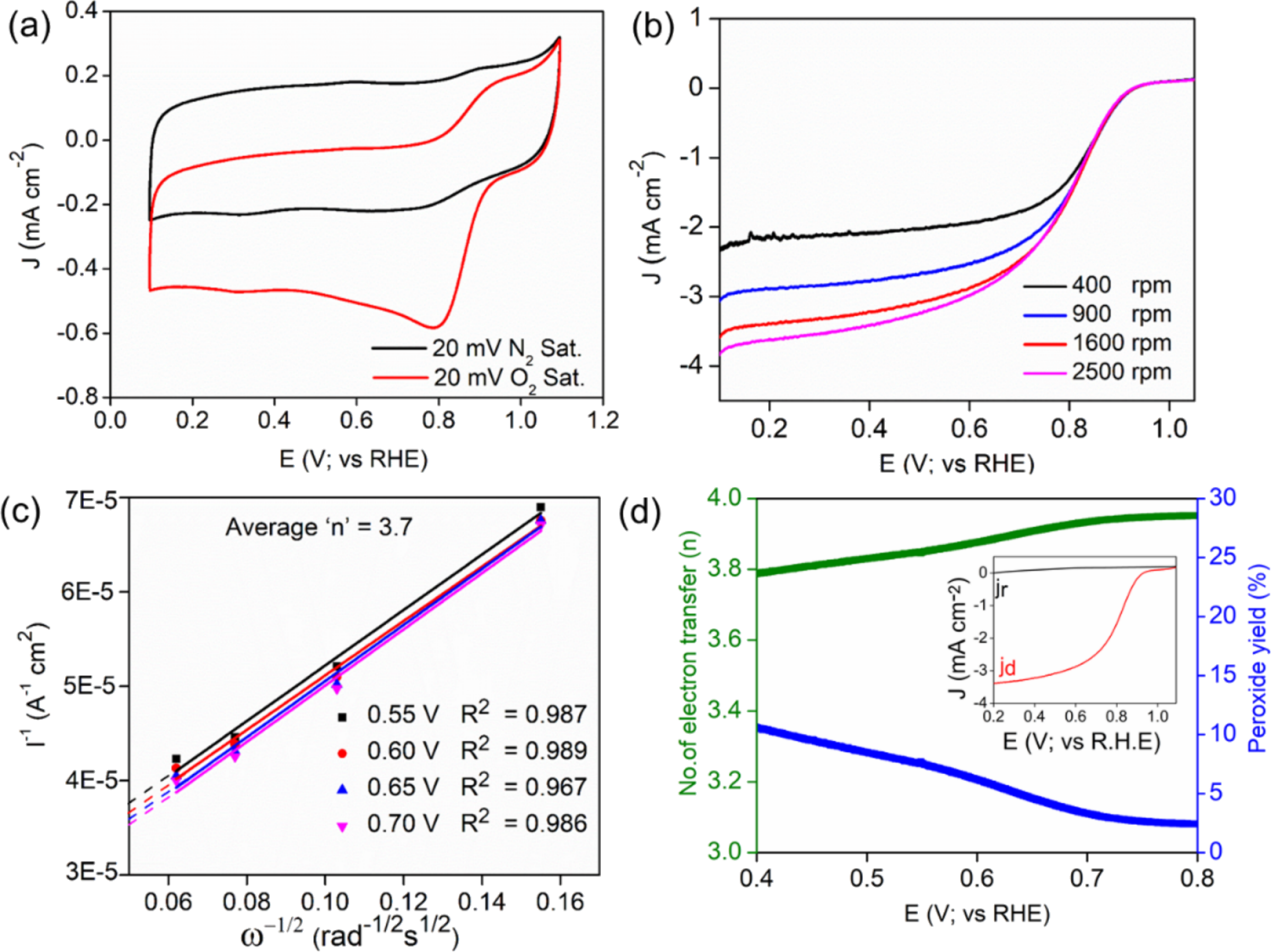
(a) Comparative CV profile of ACC-2 in N_2_- and O_2_-saturated 0.1 M KOH at a scan rate of 20 mV·s^−1^. (b) LSV curves of ACC-2 at different rotation speeds with a sweep rate of 10 mV·s^−1^. (c) Koutecky–Levich plot and (d) peroxide yield and *n* during ORR on ACC-2, the inset shows a RRDE voltammogram at a scan rate and rotation speed of 10 mV·s^−1^ and 1600 rpm, respectively.

In order to examine the ORR activity of the bi- and trimetallic electrocatalysts (Figure S5, Supporting Information File 1) under alkaline (0.1 M of KOH solution) conditions, we have performed cyclic voltammetry (CV) and linear sweep voltammetry (LSV) on a rotating disc electrode (RDE), which provides the direct chemical identification of species exposed to the electrolytic solution. In particular, the comparative CV curves for the most active ACC-2 electrocatalyst measured in 0.1 M KOH solution saturated with N_2_ or O_2_ was examined (Figure 3a). The plot shows a clear and characteristic cathodic ORR peak at 0.8 V (vs RHE), for the solution saturated with O_2_, which is not observed in N_2_-saturated solution. This indicates that ACC-2 has a superior electrocatalytic activity towards ORR in alkaline media in comparison to the other samples. For more insight into the oxygen electroreduction kinetics, ORR measurements were carried out at different rotating speeds from 400 to 2500 rpm. Figure 3b shows an onset potential of 0.94 V vs RHE with a considerable limiting current density (*J*_L_) of 3.6 mA·cm^−2^ at 1600 rpm under alkaline conditions. Particle size, composition, and exposure to active sites of the catalyst are important features of ORR electrocatalysis that need to be optimized to obtain the highest mass activity and electron transfer value (*n* ≈ 4). Oxygen reduction on smaller catalyst particles favours the two-electron pathway, dominated via active edge and corner sites, while the four-electron pathway is catalysed by larger particles [18,30]. The kinetics of oxygen reduction on the surface of the ACC-2 sample was studied via the Koutecky–Levich (K–L) method using Equation S1, Supporting Information File 1. The K–L plot (*I*^−1^ vs ω^−1/2^) obtained from the LSV curves in Figure 3b shows the linearity at different potentials (Figure 3c). Moreover, RRDE studies were carried out to measure the hydrogen peroxide generated during the electroreduction of oxygen and to get further insight into the ORR kinetics. The obtained ring current density (*J*_r_) for the ACC-2 catalyst was much smaller than the disc current density (*J*_d_) (inset of Figure 3d). The peroxide yield was found to be less than 15% for the potential range of 0.1 to 0.8 V vs RHE, whereas *n* was found to be ca. 3.7 (Figure 3d) indicating single-step ORR kinetics. Thus, our RDE and RRDE studies reveals that ACC-2 catalyst exhibits a one-step conversion of O_2_ to OH^−^ in alkaline electrolytic environment with kinetics similar to that of commercial Pt/C catalysts.

**Figure 4:**
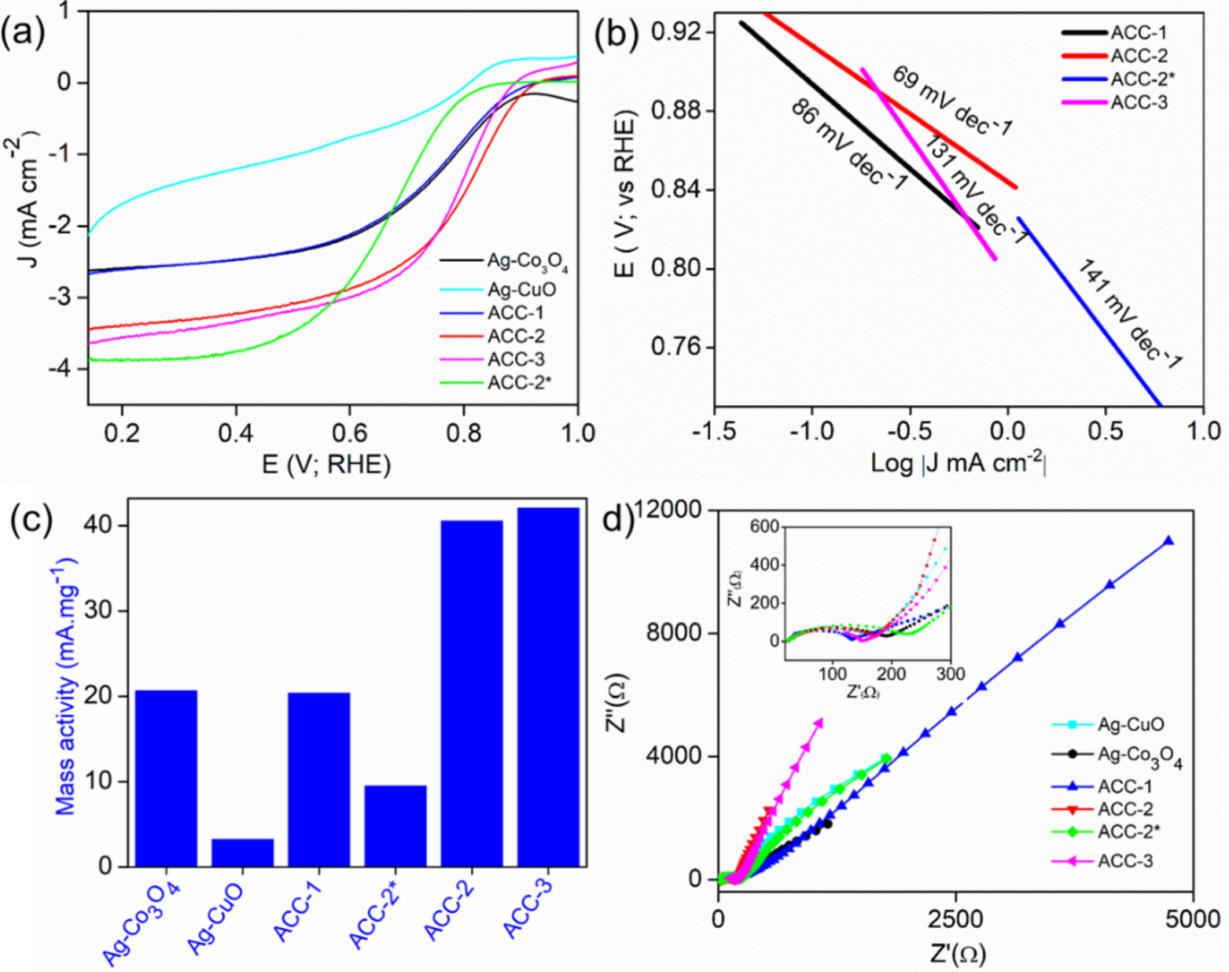
(a) Comparative ORR polarization curves of various catalysts in O_2_-saturated 0.1 M KOH electrolyte at 1600 rpm and a sweep rate of 10 mV·s^−1^. (b) Corresponding Tafel plots. (c) Mass activity obtained at 0.7 V for all active catalysts and (d) Nyquist plots (the inset shows high-frequency EIS curves).

Furthermore, the relative ORR performance of the series of prepared catalysts was evaluated using LSV measurements in O_2_-saturated 0.1 M KOH solution (Figure 4a). The catalytic performance for oxygen electroreduction was evaluated via various parameters from the ORR polarization curves including onset potential, half-wave potential, and diffusion limiting current density (*J*_L_). ACC-2 has the highest positive onset potential of the electrocatalysts. The decreasing order of onset potential is ACC-2 (0.94 V) > ACC-3 (0.92 V) > ACC-1 (0.91 V) > Ag-Co_3_O_4_ (0.90 V) > Ag-CuO (0.86 V) > ACC-2^*^ (0.85 V). The kinetics of all trimetallic electrocatalysts (both supported and supportless) were evaluated with the help of Tafel plots (Figure 4b). The lower Tafel slope of the ACC-2 sample (69 mV·dec^−1^) showed better electrokinetics than that of ACC-1 (86 mV·dec^−1^), unsupported ACC-2^*^ (141 mV·dec^−1^), and ACC-3 (131 mV·dec^−1^). Figure 4c reveals the comparative mass activity values for various catalyst materials calculated at 0.7 V, among which ACC-2 and ACC-3 exhibited significantly higher mass transport than the other electrocatalysts as shown in Table S2, Supporting Information File 1. It is important to mention that the considerably lower mass activities of our catalysts with respect to benchmark catalysts could be underutilized active sites due to the larger size and the calculation of the mass activity via normalizing *J*_k_ to the total weight of the active catalyst (including the rGO support).

In order to probe the multiple processes occurring at the electrode/electrolyte interface during the ORR, electrochemical impedance spectroscopy was performed. The Nyquist plot was acquired at 5 mV as AC amplitude between 100 kHz and 100 mHz with 0 V as a bias potential (Figure 4d). It is important to note that the prepared electrocatalysts displayed a sharp slope in the low-frequency region, representing little ionic diffusion resistance from the 0.1 M KOH solution to the Ag electrode surface throughout the ORR process (Figure 4d). The excellent electrocatalytic ORR activity of ACC-2 may originate from the composition and synergistic effects of trimetallic atoms finely dispersed over rGO. Moreover, based on the d-band centre theory, the low value of Ag impacts the low oxygen absorption energy, which can be boosted by doping with copper atoms on the surface [31]. The efficiency of the ORR depends on the design and composition of the catalyst for optimal binding with oxygen moieties and stabilizing the intermediates [21,32]. The probable oxygen reduction reaction occurring over trimetallic oxide systems under alkaline solution based on several literature reports is represented in Figure S6, Supporting Information File 1. It is divided into four steps. First, O_2_ gas gets adsorbed on the catalyst surface and is partially reduced to OOH^−^ through the first electron transfer (Figure S6, Equation S1, Supporting Information File 1). It further gets reduced via three-electron transfer (Figure S6, Equation S2 and Equation S3, Supporting Information File 1) yielding OH^−^ (Figure S6, Equation S4, Supporting Information File 1).

Additionally, we have performed a series of CV measurements at a low scan rate in order to calculate the ECSA using the double-layer capacitance method (Figure S7, Supporting Information File 1). The value of the double-layer capacitance is proportional to the active surface area of the electrode. ACC-3 displayed a comparatively high ECSA (81.8 m^2^·g^−1^), followed by ACC-2 (66.9 m^2^·g^−1^). Bimetallic Ag-Co_3_O_4_ showed 58.9 m^2^·g^−1^ and rest of the catalysts presented considerably lower ESCA values (Table S2, Supporting Information File 1). The superior ECSA values of our electrocatalysts are comparable to that of AgCo/electrochemically reduced graphene oxide and AgPdPt nanotubes (Table S3, Supporting Information File 1). Even though ACC-3 displayed a slightly higher ESCA and mass activity as compared to ACC-2, the higher onset potential and lower Tafel slope (giving rise to better electrokinetics) are the main reasons for the superior ORR performance of the ACC-2 sample.

In addition to the catalyst size, morphology, conductivity, and the exposure of active sites, the surface wettability of the electrocatalyst significantly governs the interaction between electrolyte and the electrode surface. We probed the water wetting ability of supported ACC-2 and supportless ACC-2^*^ by measuring the water contact angles (Figure S8a,b, Supporting Information File 1). The rGO-supported ACC-2 material showed a higher water wettability (14 ± 1°) than the supportless ACC-2^*^ (40 ± 5°). The improved wettability can enhance the charge transfer rate between the electrolyte and the electrode, along with enabling effective electrical integration to reduce ohmic losses, boosting the ORR activity of ACC-2. We observed that ACC-2 displayed superior ORR activity and considerable mass transport activity. Thus, it is very important to understand the microstructure and bonding information of the resultant hybrid.

**Figure 5:**
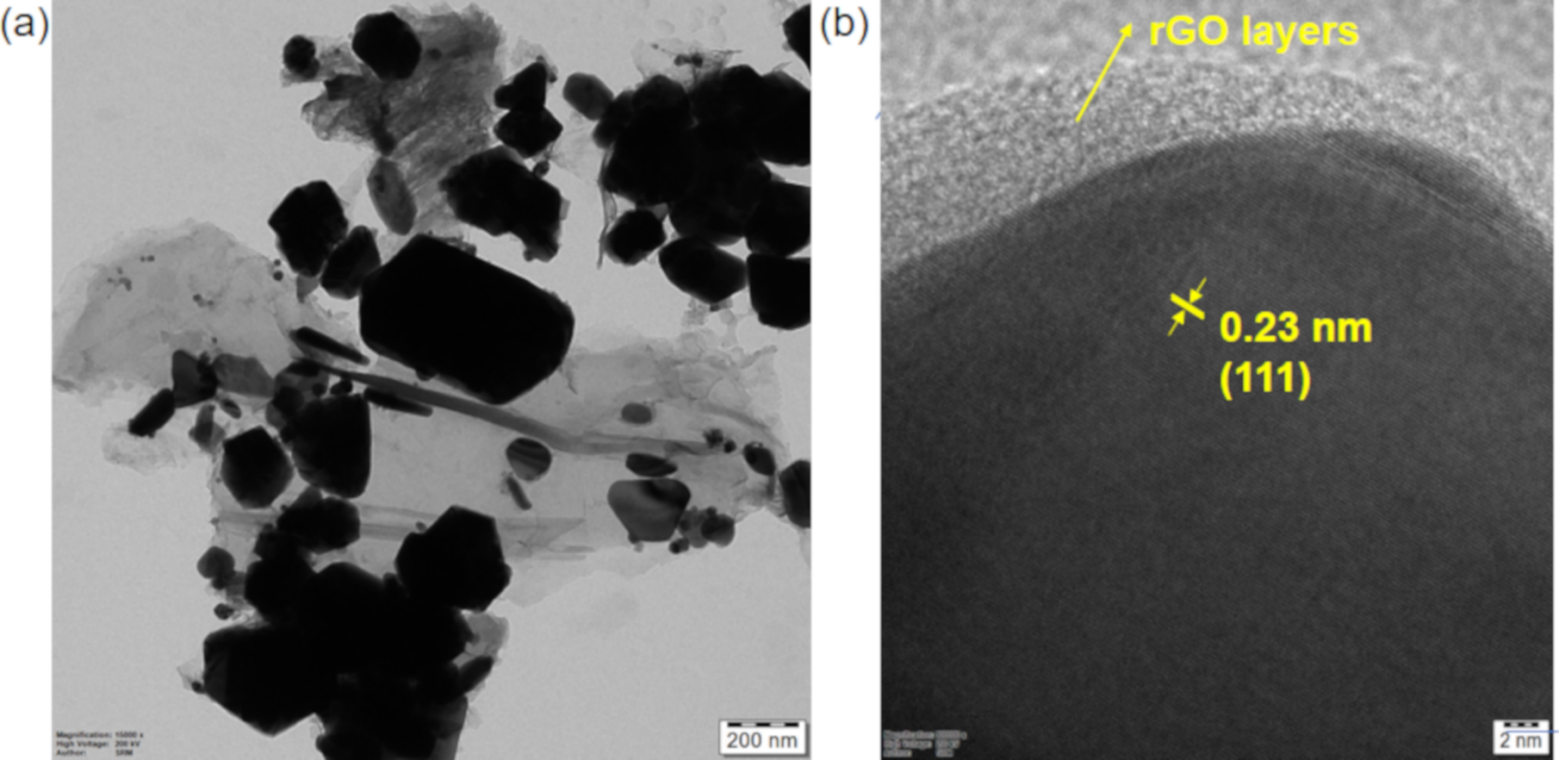
(a) TEM and (b) HRTEM images of ACC-2.

We further examined the morphology of ACC-2 and its distribution over rGO nanosheets via transmission electron microscopy (TEM). We notice a wide size distribution of the particles (100–800 nm, Figure 5a and Figure S9, Supporting Information File 1). It is important to mention that we do not see the particles outside the rGO nanosheets, despite their large sizes and the sonication during sample preparation. This is because of the functional groups with strong affinity and the scaffolding nature of rGO anchoring trimetallic NPs. The large particle sizes of the trimetallic system could be due to the slower reduction and prolonged nucleation of the constituents. The heterogeneous nature of the electrocatalyst surface is clearly evident from TEM investigation. Specifically, upon closer examination, lattice fringes of 0.23 nm, corresponding to contracted Ag(111) planes, were observed from HRTEM analysis (Figure 5b). The trimetallic NPs are tightly bound to rGO sheets, which helps to increase the oxygen reduction activity.

**Figure 6:**
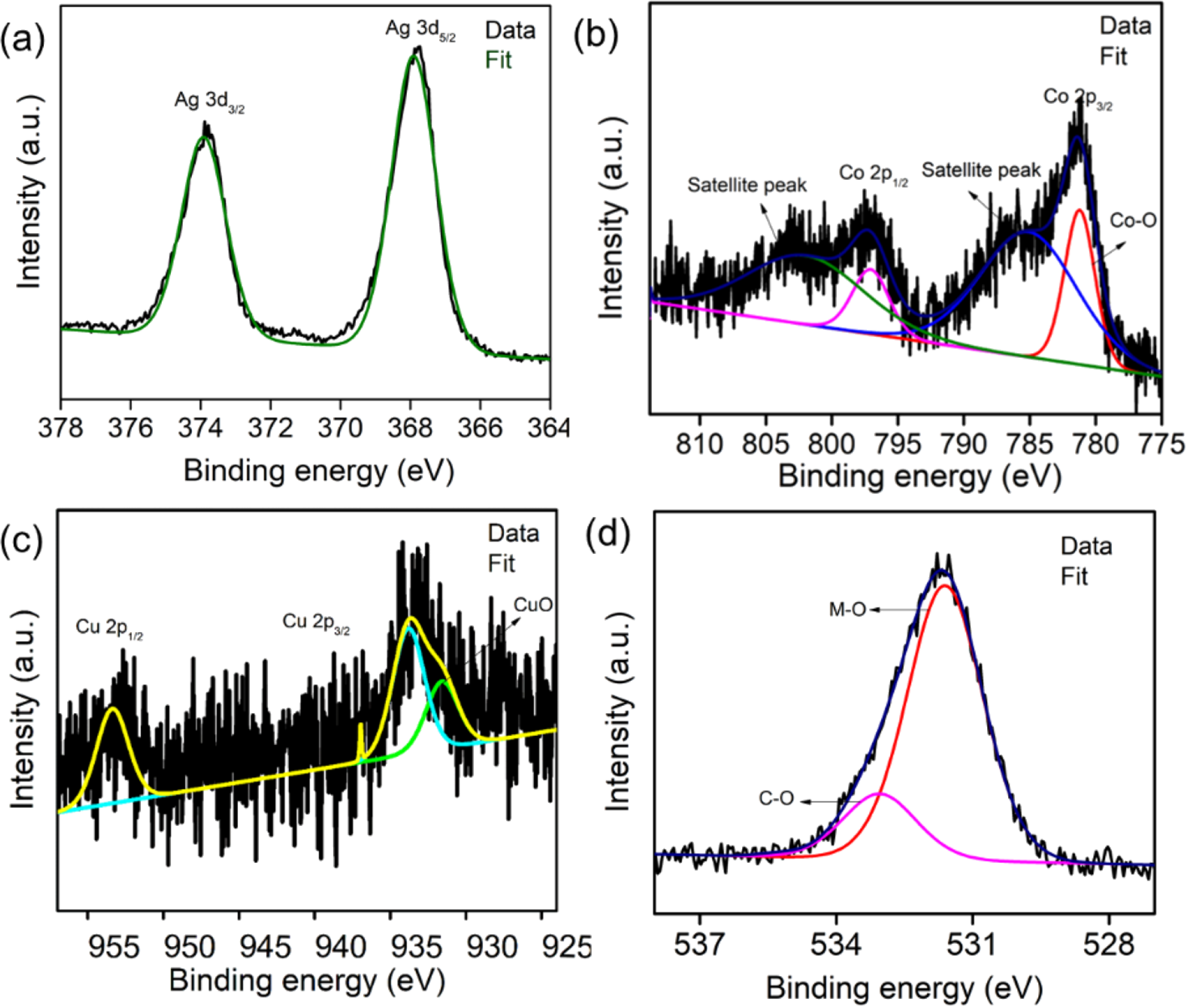
High-resolution XP spectra (a) Ag 3d, (b) Co 2p, (c) Cu 2p, and (d) O 1s of ACC-2.

We enumerate the important binding sites, oxidation states, and the elemental composition using X-ray photoelectron spectroscopy. The survey scan of ACC-2 revealed C, O, Cu, Co, and Ag with 54.7, 29.9, 1.0, 8.1, and 6.3 atom % respectively (Figure S10a, Supporting Information File 1). The Ag 3d_3/2_ and Ag 3d_5/2_ peaks in the high-resolution spectrum lie at 367.9 and 373.9 eV, respectively, with a splitting of 6 eV (Figure 6a), indicating the elemental oxidation state of Ag metal [28,33]. The Co 2p high-resolution spectrum has two primary peaks assigned to 2p_1/2_ and 2p_3/2_ at 797.3 and 781.3 eV, respectively, as well as two satellite peaks at 802.2 and 785.1 eV (Figure 6b). The peak difference between 2p_1/2_ and 2p_3/2_ and the satellite peak assignment represent the existence of cobalt atoms in Co^2+^ and Co^3+^ chemical states [34]. Likewise, the high-resolution spectrum of copper displayed Cu 2p_1/2_ and Cu 2p_3/2_ binding energies at 953.3 and 933.8 eV, respectively, with a peak at 933.1 eV (Figure 6c) for Cu–O, showing that Cu is in the +2 oxidation state. The main O 1s peak can be deconvoluted into two peaks at 533.06 and 531.62 eV, where the oxygen coordinated with metal is responsible for the major peak and the minor corresponds to the O–C linkage (Figure 6d). The peaks at 286 and 284.7 eV (Figure S10b, Supporting Information File 1) signify the graphitic nature of the carbon and C–O functional groups, respectively, which come from the supporting matrix.

**Figure 7:**
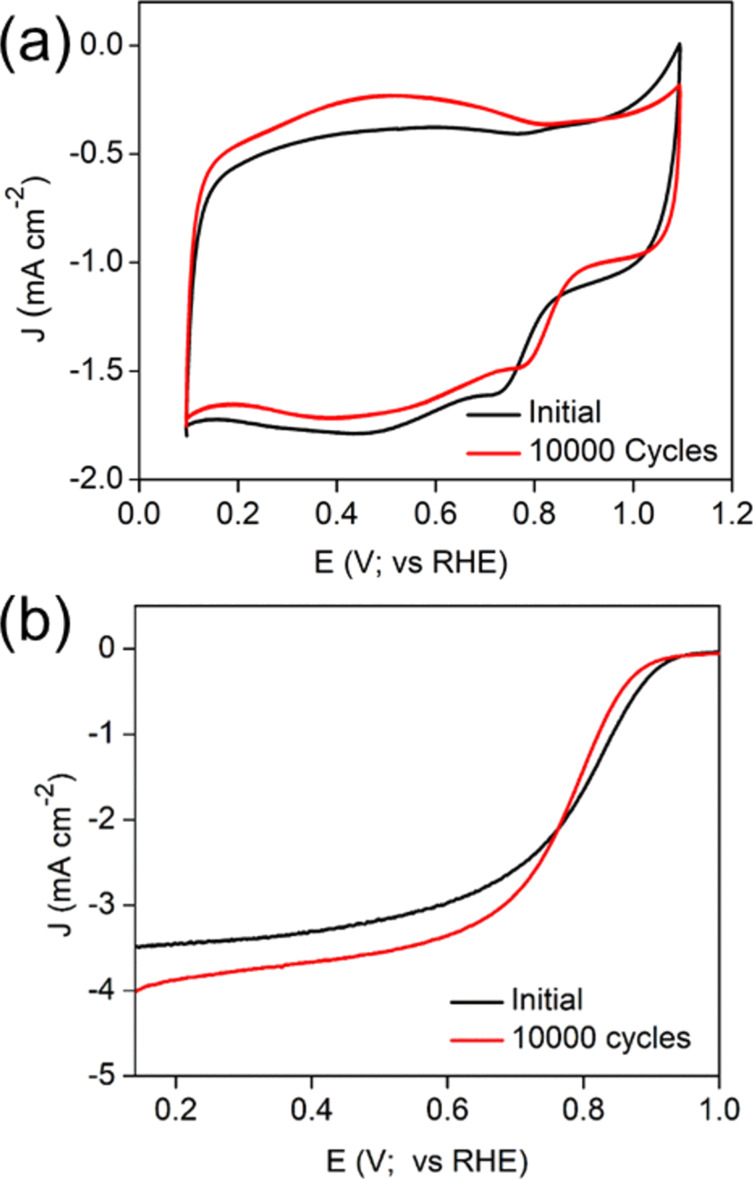
Stability of ACC-2. (a) CV curves and (b) LSV curves before and after 10,000 continuous cycles in O_2_-saturated 0.1 M KOH electrolyte.

After successfully analysing the morphology and the active sites of ACC-2, we probed the stability of the ACC-2 under alkaline conditions by continuous cycling (up to 10,000 cycles) in O_2_-saturated 0.1 M KOH solution (Figure 7a). Importantly, the ACC-2 catalyst exhibited only a slight negative shift in its half-wave potential after the 10,000 cycles stability test as shown in Figure 7b.

Following the stability investigation, another assessment of functional groups and morphology of ACC-2 was carried out by FTIR and SEM (Figure S11, Supporting Information File 1). There is no considerable change in the morphological integrity of the catalyst (before and after 10,000 cycles), highlighting the robustness of the sample (Supporting Information File 1, Figure S11b,c). The rGO framework was found to effectively maintain the operational stability of the trimetallic oxide NPs. We compared the performance of our catalyst with that of existing catalysts and emphasize the importance of synthesis conditions. Our catalysts were synthesised in a reasonably short time and displayed good ORR activity in alkaline media at low temperatures, compared to other reported Ag-based catalysts (Table S3, Supporting Information File 1).

## Conclusion

We have reported a single-step growth and assembly of bimetallic (Ag-Co_3_O_4_ and Ag-CuO) and trimetallic (AgCuCo) oxide particles dispersed on a rGO support by microwave-assisted synthesis. The morphology, composition, and functional groups were methodically analysed using various microscopic and spectroscopic techniques. Changing the silver concentration in the trimetallic oxides, in combination with conductive rGO, plays a key role in the ORR activity in alkaline medium. We optimized the Ag content in the trimetallic oxide ACC-2, which showed an onset potential of 0.94 V vs RHE and a limiting current density of 3.6 mA·cm^−2^ along with considerable ECSA (66.92 m^2^·g^−1^) and mass activity (40.55 mA·mg^−1^). The optimum electrocatalyst shows considerable electrochemical stability over 10,000 cycles in 0.1 M KOH solution. We envisage potential applications of this sustainable trimetallic catalyst and rGO-supported electrode materials for oxygen electrocatalysis.

## Supporting Information

File 1 Experimental, materials, characterization data, electrochemical measurements, water contact angle measurements, and comparison of reported ORR activities of Ag-based catalysts.
