# Supplementary material for: Electrocatalytic oxygen reduction activity of AgCoCu oxides on reduced graphene oxide in alkaline media
Source: Beilstein J Nanotechnol. 2022 Sep 26;13:1020–9. doi: 10.3762/bjnano.13.89 (PMC9531560; doi:10.3762/bjnano.13.89)
Supplement: File 1 — Experimental, materials, characterization data, electrochemical measurements, water contact angle measurements, and comparison of reported ORR activities of Ag-based catalysts. [file Beilstein_J_Nanotechnol-13-1020-s001.pdf]

BEILSTEIN JOURNAL OF NANOTECHNOLOGY

Supporting Information

for

Electrocatalytic oxygen reduction activity of AgCoCu oxides on reduced graphene oxide in alkaline media

Iyyappan Madakannu, Indrajit Patil, Bhalchandra Kakade
and Kasibhatta Kumara Ramanatha Datta

Beilstein J. Nanotechnol. **2022,** *13,* 1020–1029. doi:10.3762/bjnano.13.89

Experimental, materials, characterization data, electrochemical measurements, water contact angle measurements, and comparison of reported ORR activities of Ag-based catalysts

License and Terms: This is a supporting information file under the terms of the Creative Commons Attribution License (https://creativecommons.org/licenses/by/4.0). Please note that the reuse, redistribution and reproduction in particular requires that the author(s) and source are credited and that individual graphics may be subject to special legal provisions.
The license is subject to the *Beilstein Journal of Nanotechnology* terms and conditions: (https://www.beilstein-journals.org/bjnano/terms)

Experimental

Materials

All chemicals and solvents were used without further purification. Graphite flakes (20 μm) and PVP (M_W ≈ 10000) were purchased from Sigma Aldrich. $Co(NO_3)_2 \cdot 6H_2O$, $AgNO_3$, $Cu(NO_3)_2 \cdot 3H_2O$, $KMnO_4$ from SISCO SRL. ethylene glycol, aqueous NH_3, concentrated H_2SO_4 and 30% H_2O_2 were purchased from FISHER scientific.

Synthesis of graphite oxide (GO)

Graphite oxide was prepared following the modified procedure reported by Tour et al. and a procedure reported based on our previous study [1,2].

Synthesis of Ag-CuO, Ag-Co_3O_4, and AgCuCo oxide NPs over rGO

Precursors of $AgNO_3$, $Cu(NO_3)_2 \cdot 3H_2O$, and $Co(NO_3)_2 \cdot 6H_2O$ were used to prepare bimetallic (Ag-CuO and Ag-Co_3O_4) and trimetallic (AgCuCo oxide) NPs over rGO. 44 mL of ethylene glycol was added to 1.25 mL of 0.1 M $AgNO_3$, 0.625 mL of 0.1 M $Cu(NO_3)_2 \cdot 3H_2O$, and 0.625 mL of 0.1 M $Co(NO_3)_2 \cdot 6H_2O$, and the solution was agitated for 10 min. To this 0.025 g PVP-10000 was added and the mixture was sonicated for 10 min followed by the addition of 1 mL NH_3. 0.01 g GO was dispersed in 5 mL of ethylene glycol under sonication for 30 min and was added dropwise to the resultant mixture. The colour of solution changed to black and was agitated for 60 min before being moved to a Teflon-lined microwave reactor (Anton-Paar Multiwave pro) for 20 min of temperature-controlled fast heating with constant stirring at 170 °C. The obtained black product was isolated via centrifugation at 10000 rpm for 15 min using ethanol, and was then dried in an oven at 60 °C for 10 h. The resultant material is henceforth denoted as $Ag_{2.0}Co_{1.0}Cu_{1.0}$ (ACC-2). The same material without using graphene

oxide as a support was labelled as ACC-2*. Likewise, the other catalysts $Ag_{0.6}Co_{1.5}Cu_{1.5}$ (ACC-1) and $Ag_{6.0}Co_{1.0}Cu_{1.0}$ (ACC-3) were prepared using 0.625 and 1.875 mL of 0.1 M $AgNO_3$ and 0.937 and 0.312 mL of 0.1 M of Cu and Co salts, respectively. Bimetallic rGO-supported NPs (Ag-Co_3O_4 and Ag-CuO) were prepared using 1.25 mL of 0.1 M Ag, Cu, and Co salts.

Materials characterization

An infrared spectrometer IR-Tracer 100 Schimadzu was used to record Fourier-transform infrared spectra (FTIR) of the prepared electrocatalysts. A powder X-ray diffractometer PANalytical X'pert3 was used to carry out powder X-ray diffraction (PXRD) measurements. The morphology studies were carried out by using a scanning electron microscope (FEI QUANTA 200) with 20 kV accelerating voltage. Transmission electron microscopy (TEM) analyses of ACC-2 were carried out by using a JEOL JEM-2100 plus microscope (Japan). X-ray photoelectron spectroscopy measurements on ACC-2 were carried out by using ULVAC-PHI, Inc; Model: PHI5000 Version Probe III. The water contact angles of ACC-2 and ACC-2* (0.5–2 μL) were measured using a KYOWA DMs-40 contact angle metre (sessile drop), half-angle technique fit, and FAMAS add-in software.

Electrochemical measurements

All electrochemical measurements were performed on an electrochemical workstation (760E, CH Instrument) using a standard three-electrode system, which comprises of a graphite rod as counter electrode, silver/silver chloride (Ag/AgCl in 3 M KCl solution) as reference electrode and catalyst-loaded glassy carbon (GC) as working electrode. The working electrode was prepared by drop casting the catalyst ink onto a surface of pre-cleaned rotating disk electrode (RDE, 3 mm in diameter) and a rotating ring-disk electrode (RRDE, 4 mm in diameter). The catalyst ink was prepared by following a procedure similar to our previous study [2]. By dispersing 4 mg of each catalyst in 1 mL of IPA solution containing 20 μL of 5 wt % Nafion,

followed by ultrasonication for 30 min. Thereafter, 4 μL of catalyst ink was drop cast on RDE. The catalyst loading on RDE-GC was maintained to be 226 μg·cm^{-2} during the electrochemical studies. The ORR performance of the catalysts was measured in O_2-saturated 0.1 M KOH solution. The cyclic voltammetry (CV) curves were obtained at a scan rate of 20 mV·s^{-1}. The linear sweep voltammetry (LSV) was performed using RDE at a scan rate of 10 mV·s^{-1} with various rotation speeds (400–2500 rpm). All measured potentials are reported versus the reversible hydrogen electrode (RHE) [3]. The onset potential was defined as the potential required for generating a current density of 0.1 mA·cm^{-2} in LSV curves. The electron transfer number was calculated with the help of the Koutecky–Levich (K-L) equation:

$$\frac{1}{J} = \frac{1}{J_k} + \frac{1}{J_d} = \frac{1}{J_k} + \frac{1}{B\omega^{1/2}} \qquad (S1)$$

$$B = 0.62nFAD_{O_2}^{2/3}\,\nu^{-1/6}C_{O_2}$$

where J is the measured current density, J_k is the kinetic diffusion current density, J_d is the diffusion current density, B is the slope, ω is the angular velocity (ω= 2πN, N is the rotation speed), n is the number of transferred electrons, F is the Faraday constant (96485 C·mol^{-1}), C_0 is the saturation concentration of O_2 (1.2×10^{-6} mol·cm^{-3}), D_{02} is the diffusion coefficient of O_2 (1.9×10^{-5} cm^2·s^{-1}), and ν is the kinematic viscosity (0.01 cm^2·s^{-1}) [3].

The number of transferred electrons and the amount of generated hydrogen peroxide were investigated using RRDE measurements. The yield of hydrogen peroxide (H_2O_2) and the number of transferred electrons (n) were determined using the following equations:

$$H_2O_2\,(\%) = 200 \times \frac{I_r/N}{Id+I_r/N}$$

$$n = \frac{4 \times I_d}{I_d+I_r/N}$$

where I_d is the disk current, I_r is the ring current, and N is the current collection efficiency of the platinum ring (N = 0.38).

Moreover, the electrochemical active surface area (ECSA) was calculated via the double-layer capacitance using the following equation.

$$\mathrm{ECSA} = C_{\mathrm{DL}}/C_{\mathrm{s}}$$

where, C_{DL} is double-layer capacitance and C_{s} represents the specific capacitance under alkaline conditions [4, 5]. Finally, the stability of the catalyst was tested by electrochemical cycling in the potential range of 0.6 and 1.0 V vs RHE in O_2-staurated 0.1 M KOH solution at a scan rate of 100 mV·s^{-1} for 10,000 cycles.

The PXRD patterns of the bimetallic Ag-Co_3O_4 and Ag-CuO assemblies over rGO show peaks located at 2θ = 38.1°, 44.2°, 64.3°, and 77.1°, which can be indexed to, respectively, the (111), (200), (220), and (311) planes of fcc Ag (JCPDS #04-0783) as shown in Figure S1. For the Ag-Co_3O_4 sample, there are additional diffraction peaks at 2θ = 32.3° and 46.3°, corresponding, respectively, to the (220) and (400) planes of the Co_3O_4 JCPDS # 74-2120.

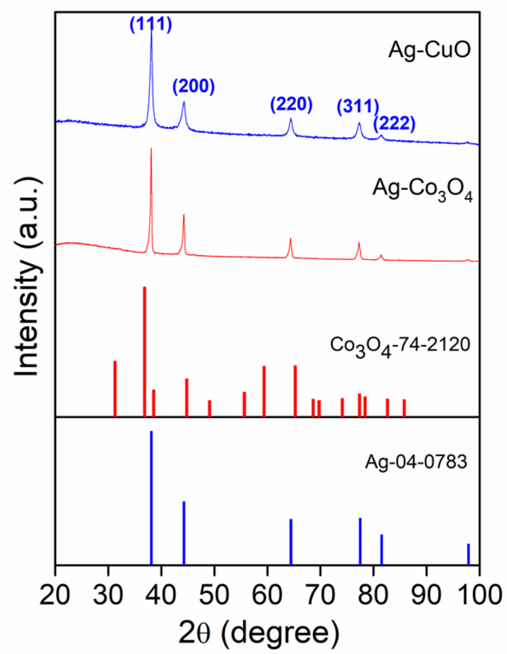

Figure S1: PXRD patterns of Ag-Co_3O_4 and Ag-CuO.

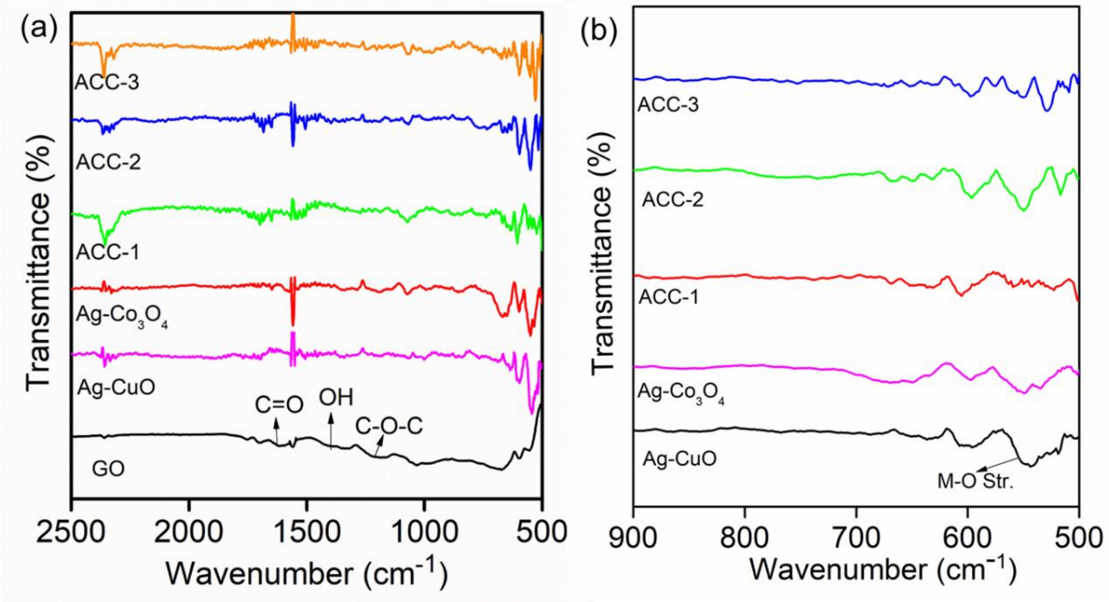

Figure S2: (a) FTIR spectra of bimetallic (Ag-CuO and $AgCo_3O_4$) and trimetallic oxide nanoparticles (ACC-1, ACC-2 and ACC-3); (b) magnified view in the region 500 to 900 cm^{-1}, representing the shift of the M–O bond of bi- and trimetallic oxides materials.

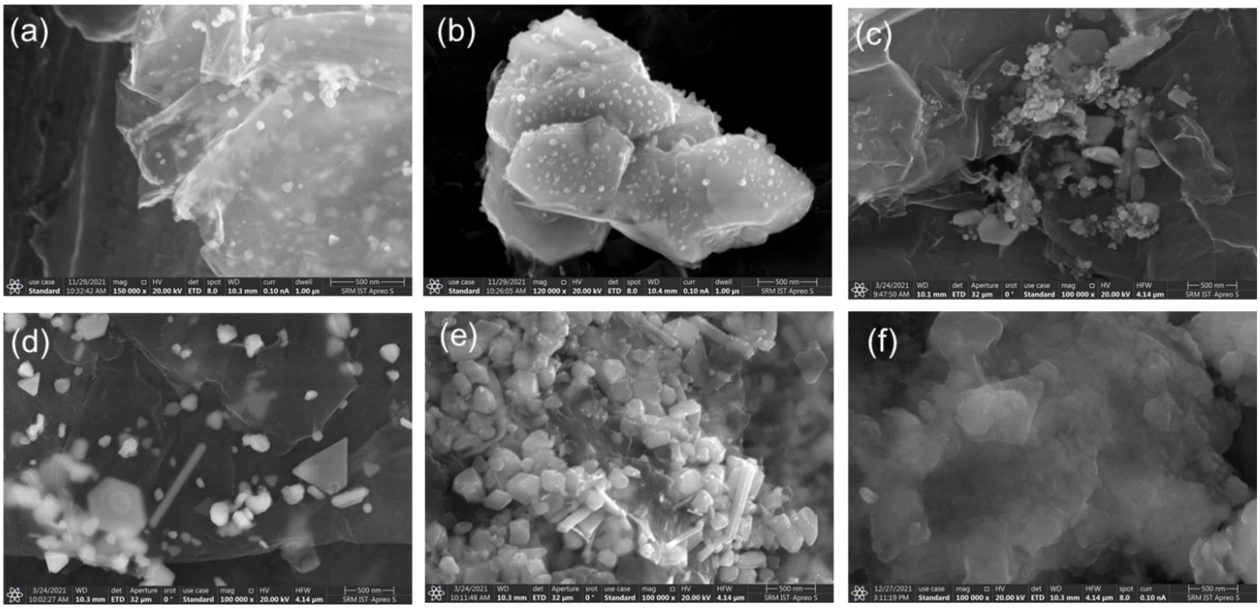

Figure S3: SEM images of (a) Ag-CuO, (b) Ag-Co_3O_4, (c) ACC-1, (d) ACC-2, (e) ACC-3, and (f) ACC-2*.

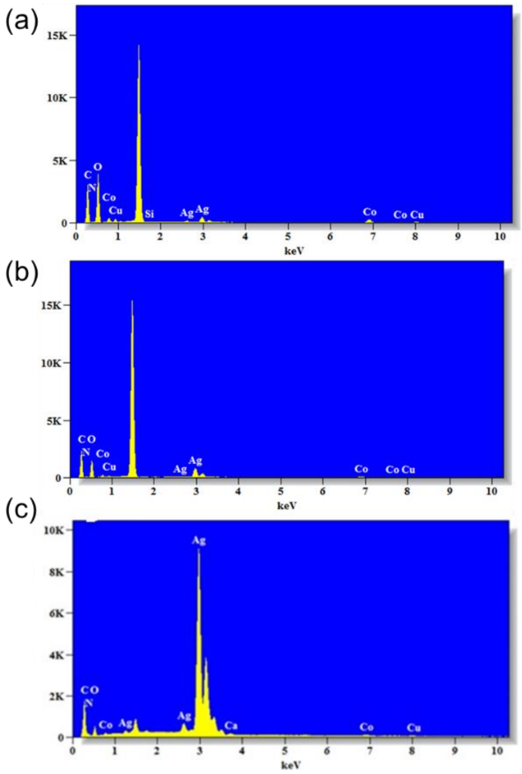

Figure S4: EDX spectra of (a) ACC-1, (b) ACC-2, and (c) ACC-3, respectively.

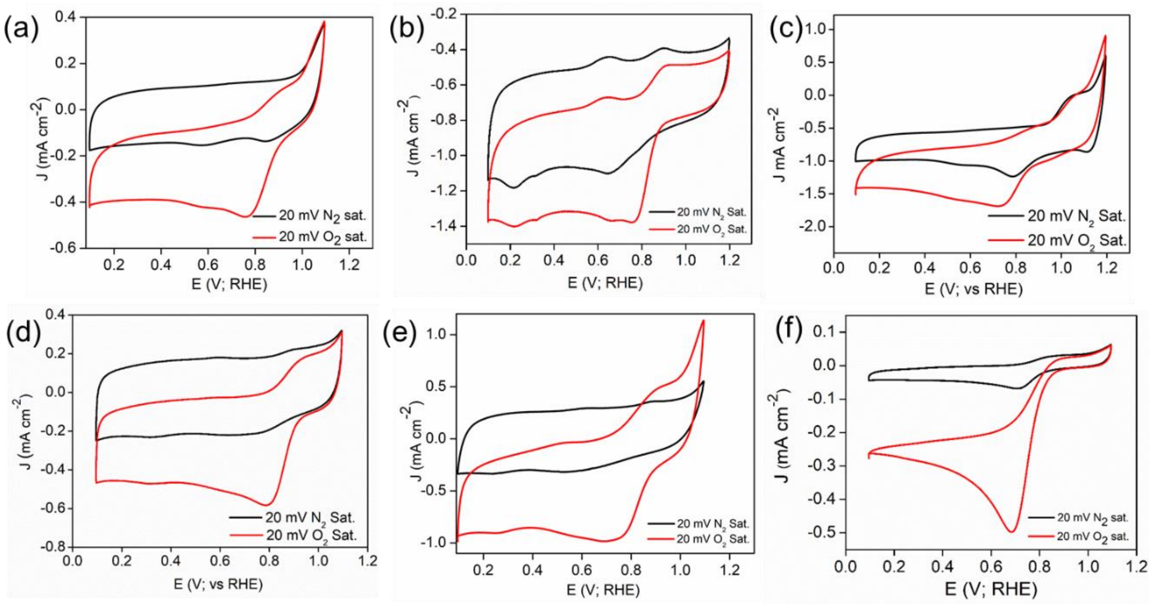

Figure S5: (a) CV profiles of prepared catalysts in N_2-saturated (black line) and O_2-saturated (red line) 0.1 M KOH at a scan rate of 20 mV·s^{-1} for (a)Ag-Co_3O_4, (b) Ag-CuO, (c) ACC-1, (d) ACC-2, (e) ACC-3, and (f) ACC-2*.

Overall reaction

$$O_{2(g)} + 2H_2O_{(l)} + 4e^{-} \longrightarrow 4\ OH^{-}$$

The reaction steps on the electrocatalyst surface are given in the following reactions:

$$O_2(g) + H_2O + 4\ e^{-} + \# \longrightarrow OOH^{\#} + OH^{-} + H_2O_{(l)} + 3\ e^{-} \quad (1)$$

$$OOH^{\#} + OH^{-} + H_2O_{(l)} + 3e^{-} \longrightarrow O^{\#} + 2\ OH^{-} + H_2O_{(l)} + 2\ e^{-} \quad (2)$$

$$O^{\#} + 2\ OH^{-} + H_2O_{(l)} + 2e^{-} \longrightarrow OH^{\#} + 3\ OH^{-} + e^{-} \quad (3)$$

$$OH^{\#} + 3\ OH^{-} + e^{-} \longrightarrow 4\ OH^{-} \quad (4)$$

#-Catalyst Surface

Figure S6: Proposed four-step ORR mechanism for ACC-2 electrocatalyst.

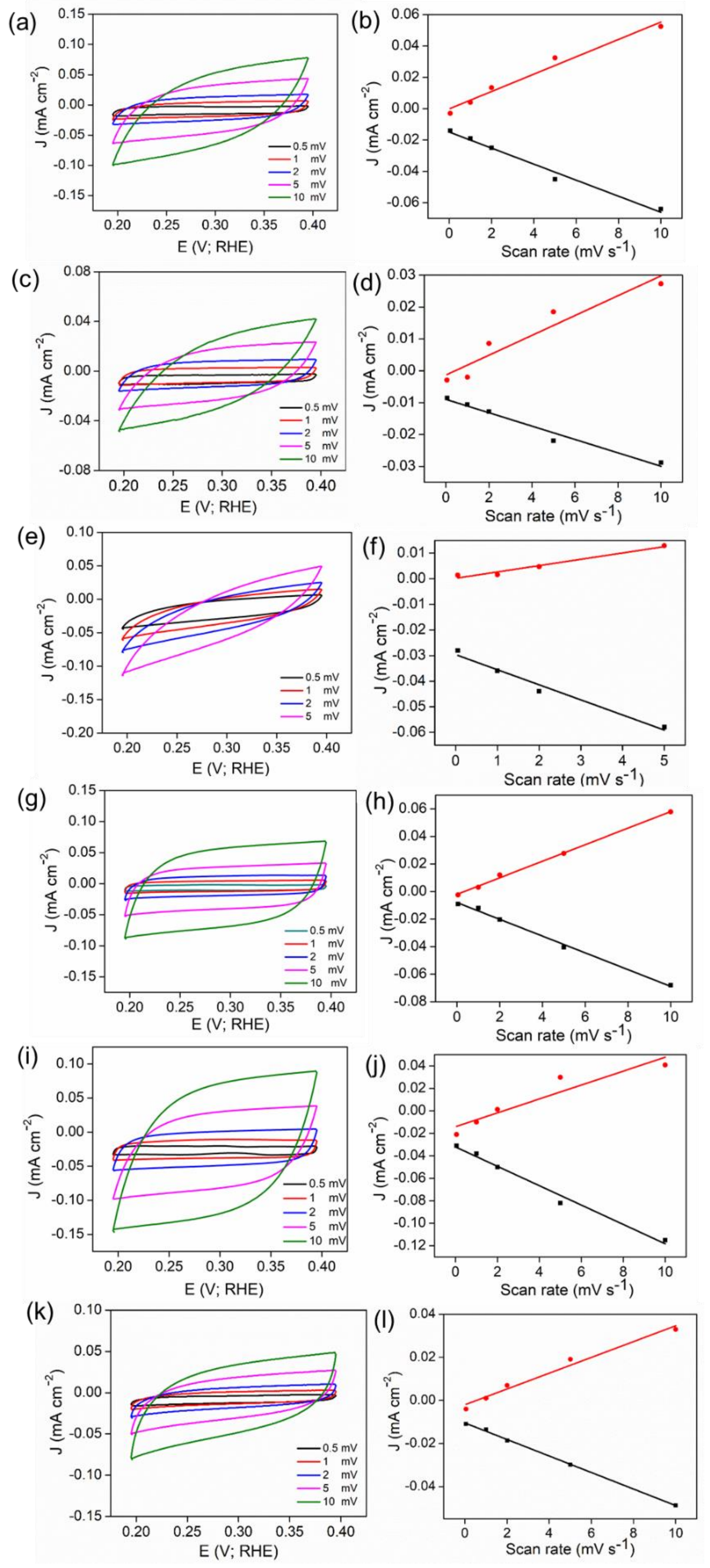

Figure S7: CV curves in a non-Faradaic region at various sweep rates (0.5, 1, 2, 5, and 10 mV·s^{-1}) in 0.1 M KOH for (a, b) Ag-Co_3O_4, (c, d) Ag-CuO, (e, f) ACC-1, (g, h) ACC-2, (i, j) ACC-3, and (k, l) ACC-2*.

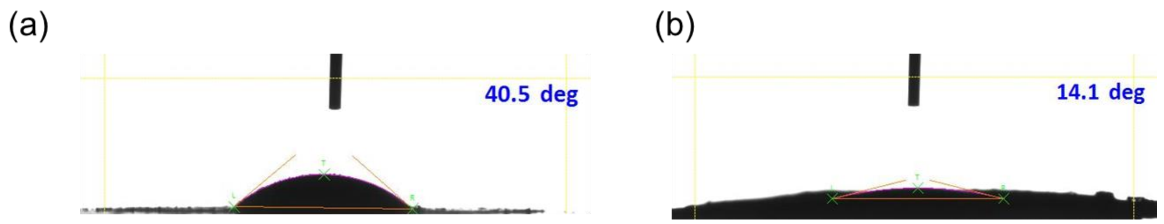

Figure S8: WCA measurements performed on (a) ACC-2* (40 ± 1°) and (b) ACC-2 (14 ± 1°).

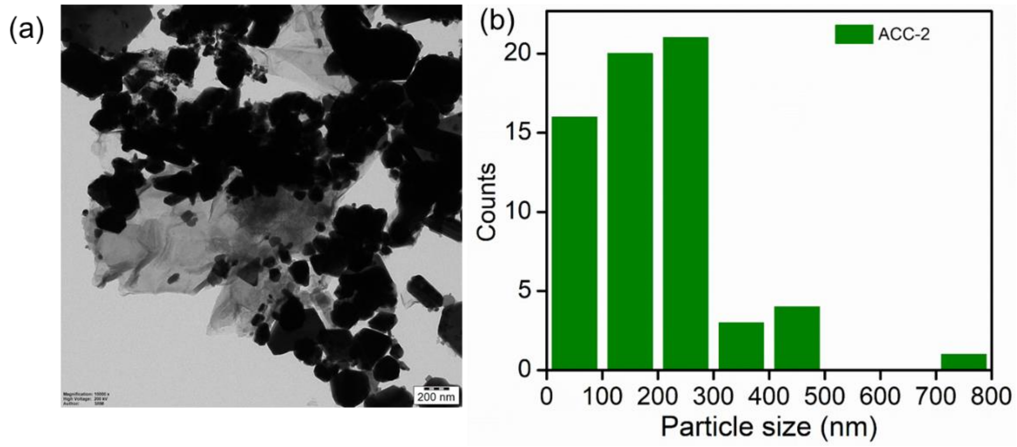

Figure S9: (a) TEM image and (b) corresponding particle size distribution of ACC-2.

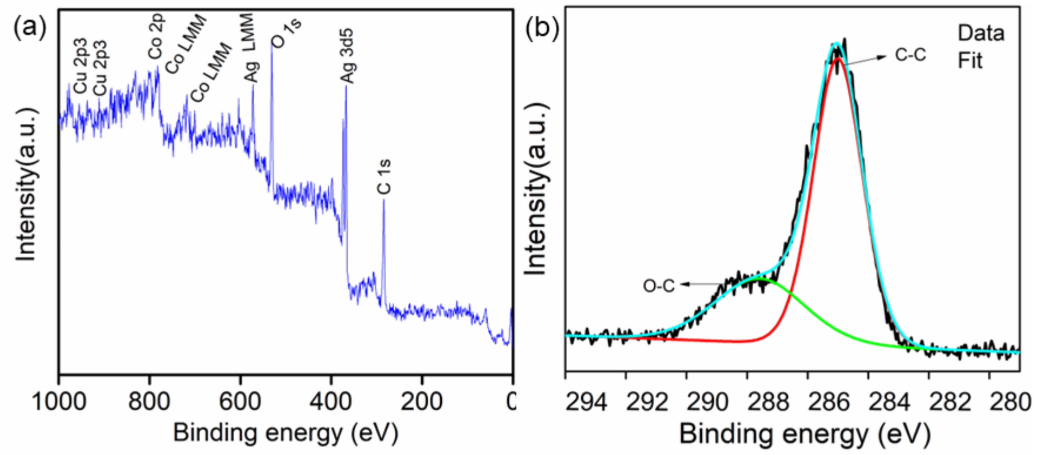

Figure S10: (a) Survey scan and (b) high-resolution C 1s XP spectra of ACC-2.

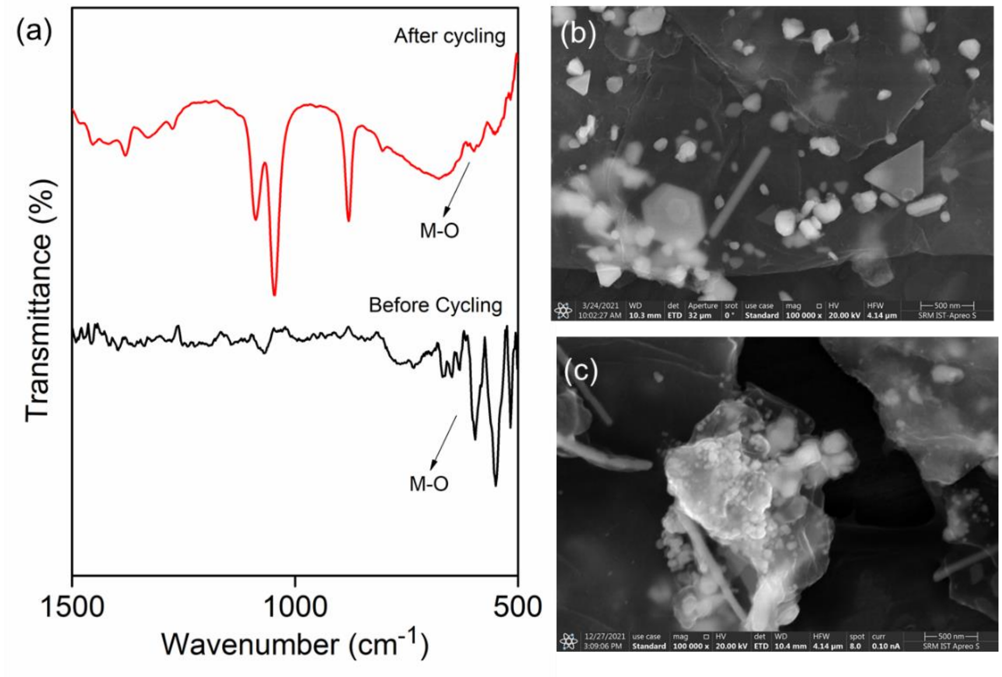

Figure S11: (a) FTIR spectra and SEM image of ACC-2 (b) before and (c) after 10,000 stability cycles.

Table S1: Particulate sizes calculated using the Scherrer equation for the bi- and trimetallic NPs over rGO.

Sl. No.	Sample name	Particulate size (nm)
1	ACC-1	61
2	ACC-2	74
3	ACC-3	60
4	ACC-2 *	65
5	Ag-Co_3O_4	31
6	Ag-CuO	11

Table S2: Comparison of ORR activity parameters (mass and ECSA) for the bi- and trimetallic NPs over rGO.

Sl. No.	Electrocatalyst	Mass activity (mA/mg)	ECSA (m^2/g)
1	ACC-1	20.38	46.23
2	ACC-2	40.55	66.92
3	ACC-3	42.05	81.80
4	ACC-2 *	9.50	41.48
5	Ag-Co_3O_4	20.66	58.96
6	AgCuO	3.25	28.76

Table S3: Comparison of ACC-2 with other synthesis techniques and key ORR parameters of binary and ternary Ag-based catalysts.

Sl. No.	Electrocatalyst	Synthetic route	Working Electrode	ORR Parameters	Stability	Ref.
1	AgCu NPs @ Ni foam	Electrochemical deposition (50 sec) of Ag-Cu NPs over Ni foam using a complexing agent	Ni foam	E_{onset} = NM<br>$E_{1/2}$ = NM<br>J_{kl} = NM<br>n = 3.9<br>Mass activity = 29 mA·mg^{-1}<br>ECSA = NM	-	[6]
2	Ag_2-Cu_1 NPs	Solution combustion method	Glassy carbon electrode	E_{onset} = 0.79 V vs. RHE<br>$E_{1/2}$ = NM<br>J_{kl} = 4.6 mA·cm^{-2}<br>n = 3.85<br>Mass activity = 38.6 mA·mg^{-1}<br>ECSA = NM	-	[7]
3	Ag_4Cu NPs	Three step route<br>Melting of the metals<br>and melt spinning followed by chemical etching by dealloying	Glassy carbon electrode	E_{onset} = 0.90 V vs. RHE<br>$E_{1/2}$ = 0.82 V<br>J_{kl} = 6 mA·cm^{-2}<br>n = 3.86<br>Mass activity = NM<br>ECSA = NM	5,000 cycles	[8]
4	AgCu/ Ordered Mesoporous Carbon (OMC)	OMC: Soft templating followed by acid etching<br>Impregnation of Ag and Cu salts followed by calcination under H_2 atm.	Glassy carbon electrode	E_{onset} = 1.00 vs. RHE<br>$E_{1/2}$ = 0.82 V<br>J_{kl} = 5.2 mA·cm^{-2}<br>n = 3.8<br>Mass activity = NM<br>ECSA = NM	20,000 sec (Chronoamperometric studies) with the loss of 10%	[9]

5	AgCo composite nanotubes	Electrospinning yielding Co^{2+}/PVP fibres. Calcination followed by chemical reduction and galvanic replacement.	Glassy carbon electrode	E_{onset} = - 0.067 V vs. SCE<br>$E_{1/2}$ = NM<br>J_{kl} = 4.75 mA·cm^{-2}<br>n = 3.80<br>Mass activity = NM<br>ECSA = NM	10,000 sec (Chronoamperometric studies) with the loss of 4%	[10]
6	AgCo alloy	Multistage incipient-wetness impregnation followed by calcination under H_2 atm.	Glassy carbon electrode	E_{onset} = 0.8 V vs. RHE<br>$E_{1/2}$ = NM<br>J_{kl} = 3.9 mA·cm^{-2}<br>n = 3.8<br>Mass activity = NM<br>ECSA = NM	10,000 cycles	[11]
7	Ag/Ag_2O @Co metallo covalent organic framework	Solvothermal heating followed by freeze drying	Glassy carbon electrode	E_{onset} = 0.87 V vs. RHE<br>$E_{1/2}$ = 0.76 V<br>J_{kl} = 4.8 mA·cm^{-2}<br>n = 2.5<br>Mass activity = NM<br>ECSA =14 cm^{-2}	40 h (Chronoamperometric studies) with the loss of 4%	[12]
8	Hollow AgPdPt nanotubes	Micelle assisted galvanic replacement followed by acid etching	Glassy carbon electrode	E_{onset} = 0.98 V vs. RHE<br>$E_{1/2}$ = 0.90 V<br>J_{kl} = 5.3 mA·cm^{-2}<br>n = 3.97<br>Mass activity = 0.61 mA μg^{-1} (normalized with respect to Pt-loading)<br>ECSA = 54.7 m^2·g^{-1}	5,000 cycles	[13]
9	AgCo-NGr	Refluxing of metal salts, GO and NH_3 followed by hydrothermal and freeze drying	Glassy carbon electrode	E_{onset} = 0.90 V vs. RHE<br>$E_{1/2}$ = 0.82 V<br>J_{kl} = 4.95 mA·cm^{-2}<br>n = 3.9<br>Mass activity = NM<br>ECSA = 9.27 m^2·g^{-1}	5,000 cycles	[14]
10	AgCo/ Electrochemically reduced graphene oxide (ERGO)	Ag, Co salts are mixed with GO followed by reducing with $NaBH_4$ Electrochemical reduction of the resultant composite.	Glassy carbon electrode	E_{onset} = -0.08 V vs. Ag/AgCl<br>$E_{1/2}$ = NM<br>J_{kl} = 5 mA·cm^{-2}<br>n = 3.85-4.0<br>Mass activity = 0.287 mA.μg^{-1} (normalized by the Ag-loading)<br>ECSA = 92 m^2·g^{-1}	10,000 sec (Chronoamperometric studies) with the loss of 4%	[15]
11	Co_3O_4/Ag @NrGO	Ag, Co salts were mixed with GO under NH_3 followed by solvothermal treatment and freeze drying	Glassy carbon electrode	E_{onset} = 0.974 V vs. RHE<br>$E_{1/2}$ = 0.735 V<br>J_{kl} = 6 mA·cm^{-2}<br>n = 3.86<br>Mass activity = NM<br>ECSA = NM	40,000 sec (Chronoamperometric studies) with the loss of 4%	[16]
12	ACC-2	Ag, Co, Cu salts are mixed with GO nanosheets under aqueous NH_3 followed by microwaving at 170 °C for 20 min	Glassy carbon electrode	E_{onset} = 0.94 V vs. RHE<br>$E_{1/2}$ = 0.78 V<br>J_{kl} = 3.6 mA·cm^{-2}<br>n = 3.7<br>Mass activity = 40.55 mA·mg^{-1}<br>ECSA = 66.92 m^2·g^{-1}	10,000 cycles	This work

NM: Not mentioned

References

1. Marcano, D. C.; Kosynkin, D. V.; Berlin, J. M.; Sinitskii, A.; Sun, Z.; Slesarev, A.; Alemany, L. B.; Lu, W.; Tour, J. M. *ACS Nano* **2010**, *4*, 4806-4814. doi:10.1021/nn1006368
2. Madakannu, I.; Patil, I.; Kakade, B. A.; Kasibhatta, K. R. D. *Mater. Chem. Phys.* **2020**, *252*, 123238. doi:10.1016/j.matchemphys.2020.123238
3. Patil, I. M.; Swami, A.; Chavan, R.; Lokanathan, M.; Kakade, B. *ACS Sustain. Chem. Eng.* **2018**, *6*, 16886-16895. doi:10.1021/acssuschemeng.8b04241
4. Govindarajan, N.; Kastlunger, G.; Heenen, H. H.; Chan, K. *Chemical Science* **2022**, *13*, 14-26, 10.1039/D1SC04775B. doi:10.1039/D1SC04775B
5. McCrory, C. C. L.; Jung, S.; Peters, J. C.; Jaramillo, T. F. *Journal of the American Chemical Society* **2013**, *135*, 16977-16987. doi:10.1021/ja407115p
6. Jin, Y.; Chen, F.; Lei, Y.; Wu, X. *ChemCatChem* **2015**, *7*, 2377-2383. doi:10.1002/cctc.201500228
7. Ashok, A.; Kumar, A.; Matin, M. A.; Tarlochan, F. *J. Electroanal. Chem.* **2019**, *844*, 66-77. doi:10.1016/j.jelechem.2019.05.016
8. Yin, S.; Shen, Y.; Zhang, J.; Yin, H.-M.; Liu, X.-Z.; Ding, Y. *Appl. Surf. Sci.* **2021**, *545*, 149042. doi:10.1016/j.apsusc.2021.149042
9. Qiao, Y.; Ni, Y.; Chen, Z.; Kong, F.; Li, R.; Zhang, C.; Kong, A.; Shan, Y. *J. Electrochem. Soc.* **2019**, *166*, H272-H282. doi:10.1149/2.0361908jes
10. Yu, A.; Lee, C.; Lee, N.-S.; Kim, M. H.; Lee, Y. *ACS Appl. Mater. Interfaces* **2016**, *8*, 32833-32841. doi:10.1021/acsami.6b11073
11. Holewinski, A.; Idrobo, J.-C.; Linic, S. *Nat. Chem.* **2014**, *6*, 828-834. doi:10.1038/nchem.2032

12. Wang, M.; Wang, C.; Liu, J.; Rong, F.; He, L.; Lou, Y.; Zhang, Z.; Du, M. *ACS Sustain. Chem. Eng.* **2021**, *9*, 5872-5883. doi:10.1021/acssuschemeng.0c09205

13. Deng, Y.; Yin, S.; Liu, Y.; Lu, Y.; Cao, X.; Wang, L.; Wang, H.; Zhao, Y.; Gu, H. *ACS Appl. Nano Mater.* **2019**, *2*, 1876-1882. doi:10.1021/acsanm.8b02206

14. Qaseem, A.; Chen, F.; Wu, X.; Zhang, N.; Xia, Z. *J. Power Sources* **2017**, *370*, 1-13. doi:10.1016/j.jpowsour.2017.10.004

15. Joo, Y.; Ahmed, M. S.; Han, H. S.; Jeon, S. *Int. J. Hydrog. Energy* **2017**, *42*, 21751-21761. doi:10.1016/j.ijhydene.2017.07.123

16. Wang, Q.; Miao, H.; Sun, S.; Xue, Y.; Liu, Z. *Chem. Eur. J.* **2018**, *24*, 14816-14823. doi:10.1002/chem.201803236
